# Molecular Mechanisms Involved in the Pathogenesis of Alphavirus-Induced Arthritis

**DOI:** 10.1155/2013/973516

**Published:** 2013-08-28

**Authors:** Iranaia Assunção-Miranda, Christine Cruz-Oliveira, Andrea T. Da Poian

**Affiliations:** ^1^Departamento de Virologia, Instituto de Microbiologia Professor Paulo de Góes, Universidade Federal do Rio de Janeiro, 21941-902 Rio de Janeiro, RJ, Brazil; ^2^Programa de Biologia Estrutural, Instituto de Bioquímica Médica, Universidade Federal do Rio de Janeiro, Avenida Carlos Chagas Filho 373, 21941-902 Rio de Janeiro, RJ, Brazil

## Abstract

Arthritogenic alphaviruses, including Ross River virus (RRV), Chikungunya virus (CHIKV), Sindbis virus (SINV), Mayaro virus (MAYV), O'nyong-nyong virus (ONNV), and Barmah Forest virus (BFV), cause incapacitating and long lasting articular disease/myalgia. Outbreaks of viral arthritis and the global distribution of these diseases point to the emergence of arthritogenic alphaviruses as an important public health problem. This review discusses the molecular mechanisms involved in alphavirus-induced arthritis, exploring the recent data obtained with *in vitro* systems and *in vivo* studies using animal models and samples from patients. The factors associated to the extension and persistence of symptoms are highlighted, focusing on (a) virus replication in target cells, and tissues, including macrophages and muscle cells; (b) the inflammatory and immune responses with recruitment and activation of macrophage, NK cells and T lymphocytes to the lesion focus and the increase of inflammatory mediators levels; and (c) the persistence of virus or viral products in joint and muscle tissues. We also discuss the importance of the establishment of novel animal models to test new molecular targets and to develop more efficient and selective drugs to treat these diseases.

## 1. Introduction

Alphaviruses are enveloped single-stranded positive-sense RNA viruses that belong to the *Togaviridae* family. They are transmitted to humans through the bite of mosquitos from the genera *Culex sp.* and *Aedes (A. albopictus *and* A. aegypti*), in a cycle involving vertebrate reservoir hosts [1, 2]. Alphaviruses are subgrouped accordingly to the prevalence of the clinical symptoms they cause in humans. The encephalitic alphaviruses occur in the Americas and are associated with severe and lethal encephalitis. This group includes the Venezuelan, Eastern, and Western equine encephalitis viruses [3]. The arthritogenic group causes incapacitating and long lasting articular disease/myalgia and comprises the Ross River virus (RRV), Chikungunya virus (CHIKV), Sindbis virus (SINV), Mayaro virus (MAYV), O'nyong-nyong virus (ONNV), and Barmah Forest virus (BFV) [2, 4]. These viruses are globally distributed and are responsible for endemic diseases in some regions (Table 1).

 Epidemiological studies on alphaviruses' infections are restricted due to insufficient surveillance and laboratory diagnostic analyses in most endemic countries, which result in an underestimation of the numbers of cases [5, 6]. Similarities between the clinical manifestations of the diseases caused by alphaviruses and those caused by others virus, such as dengue virus (a member of the *Flaviviridae* family) or Oropouche virus (a member of the *Bunyaviridae* family), also make the diagnosis difficult [7, 8]. This is especially frequent in the case of MAYV infections, in which the limited diagnosis of cases makes the illness largely unknown [6, 8, 9]. Studies on CHIKV infection were also limited before the epidemics at the La Réunion Island, a French territory in the southwest Indian Ocean, where more than 200,000 habitants were infected between 2005 and 2007 [10, 11]. In this outbreak, more than 50% of CHIKV-infected adults presented a severe disease with persistent joint pain [12–14]. After this CHIKV epidemics, several other cases of CHIKV infection were described in many countries and systematic efforts on the investigation of the pathogenesis of CHIKV infection allowed a rapid increase in the knowledge regarding the disease [11, 15, 16]. In contrast, epidemics of ONNV infection, which promote a disease similar to that caused by CHIKV, have been described in Africa since 1959, although ONNV and the pathogenesis of its infection have remained unstudied so far [17, 18]. The outbreaks of RRV, SINV, CHIKV, and some descriptions of MAYV cases are nowadays considered sufficient to point the emergence or reemergence of arthritogenic alphaviruses as an important public health problem with challenges on vector control and development of new strategies to prevent and treat these diseases [19, 20]. 

In this review, we aimed at discussing the molecular mechanisms that may be associated with exacerbation of muscular/articular damage and with the establishment of arthritis as well as the persistence of symptoms of the alphavirus infection, exploring recent data obtained with *in vitro* systems and *in vivo* studies using animal models and samples from patients.

## 2. Alphavirus-Induced Arthritis

Arthritogenic alphaviruses usually cause an acute disease, with the onset of symptoms after 3–10 days after infection, and a short (4–7 days) viremia period [18, 21–23]. The clinical manifestations include fever, headache, rash, fatigue, arthritis, arthralgia, and muscular pain [4]. Rash occurs in over 40% of the cases and may appear before, simultaneously or after arthralgia symptoms, lasting 7–10 days [23–26]. Fever can be absent in some cases, mainly in SINV, RRV, and BFV infections [21, 26, 27]. Arthritis is the most prevalent among the symptoms, with the recovery from pain and swelling occurring after some days of infection, although several reports describe the persistence of joint manifestations for months or even years [2, 19, 22, 28–31]. Joint pain and inflammation mainly affect symmetrically the small joints (such as those from fingers, wrists, and tarsus), but eventually occur in the large joints (such as those from knees and shoulders) and may also involve several joints simultaneously (polyarthralgy/polyarthritis) [13, 21, 29, 30]. Besides rash and arthritis, myalgia is a very common symptom during alphaviruses infection, demonstrating also the virus tropism for the muscular tissue [32]. 

Cellular inflammatory infiltration in joint, muscle, and associated tissues during alphavirus infection has been reported in some mouse models of RRV, SINV, and CHIKV infection, suggesting that muscular and articular damage is an immunopathological inflammatory disorder [33–35]. In RRV and CHIKV infection, the cellular infiltrate reaches synovial tissue, which shows a strong hyperplasia [34, 36, 37]. Monocytes, macrophages, NK cells, and CD4^+^ and CD8^+^ T lymphocytes are the main cellular components of the inflammatory infiltrate in animal models, indicating an involvement of these cells in the pathogenesis of the arthritis induced by alphaviruses [34, 36–38]. In agreement with the data obtained in animal models, macrophages and NK cells have been detected in synovial exudates from RRV infected patients [39–41], and a pronounced increase in the plasma levels of inflammatory mediators as well as a high CD8^+^ T lymphocyte activation were found in CHIKV patients in the acute phase of infection [42]. Furthermore, an isolated strain of CHIKV from La Réunion epidemics was able to induce a marked swelling of the hind foot in 6-week-old mice 7 days after local subcutaneous injection, which is consistent with the rheumatic symptoms observed in humans [37]. 

Chronic arthralgia and arthritis due to alphavirus infection cause clinical manifestations ranging from only a restriction of movements with persistence of swelling and pain to a severe and incapacitating disease [14, 28, 29, 43, 44]. Several studies in which patients infected with CHIKV were accompanied for long periods after La Réunion epidemics consistently demonstrated the chronic and severe manifestation of disease [14, 31, 43, 45]. Also, long lasting myalgia, arthralgia, and arthritis occur in about 25–55% of patients infected with RRV, SINV, and CHIKV [14, 30–32, 45–47]. In BFV infection, duration of symptoms seems to be reduced, and MAYV infection is very poorly described in the literature [26]. The causes of the persistence of symptoms remain inconclusive but seem to be associated with the intensity of the inflammatory process, the extension of articular lesion, and the presence of viral products in the joint tissue, as well as due to an autoimmunity process [4, 48].

## 3. Pathogenesis of the Arthritis Caused by Alphaviruses

**Figure 1:**
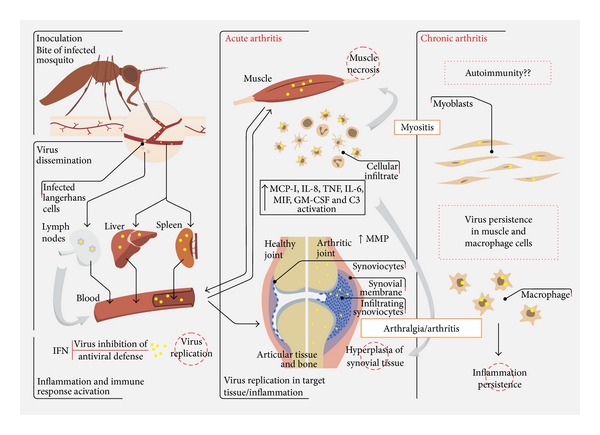
*Pathogenesis of alphavirus-induced arthritis/myositis*. After inoculation through the bite of an infected mosquito in the skin, alphaviruses disseminate in the host organism through the bloodstream. Liver, spleen, muscle, and lymph nodes are sites of primary replication, allowing an efficient virus spread. Langerhans cells facilitate virus delivery to the lymph nodes. Interferon (IFN) program is early activated, but the alphaviruses developed several mechanisms to inhibit this antiviral response. The acute phase of the disease involves virus replication followed by an inflammatory response in the target tissues, which is characterized by an extensive infiltration of lymphocytes, NK cells, neutrophils, and macrophages (the main component). The increase in the levels of several proinflammatory cytokines and chemokines in the site of infection and in the plasma is associated with myositis and arthralgia/arthritis. Also, the secretion of metalloproteinases (MMP) in the joint tissue may contribute to articular damage. Persistence of the symptoms may be related to the persistence of the virus or its products in the target cells with the subsequent accumulation of inflammatory mediators such as IL-6 and GM-CSF. A question that remains open is whether an autoimmune process is associated to the persistence of the inflammatory response, as observed for rheumatoid arthritis.

After subcutaneous inoculation by the mosquito bite, alphaviruses seem to be disseminated in the host through the lymph nodes route and the microvasculature (Figure 1). Leukopenia in acute phase of the disease is a very common hematologic alteration in alphavirus infection, suggesting a primary replication of the virus in the leukocytes [19, 49, 50]. Liver and spleen are also considered sites of primary viral replication and contribute to virus dissemination [51]. After dissemination, the virus reaches bones, muscles, and articular tissues, generating the acute phase of the disease, which is strongly associated with a local inflammatory process [34–37, 52]. Host age, the status of the immune system, virus strain virulence, and viral persistence are key determinants for the pathogenesis of alphavirus infection in animals [37, 53, 54]. For example, mice susceptibility to SINV-infection seems to involve age-dependent inflammation associated with stress response to infection [55–58].

Disease severity and persistence of symptoms are associated to the extension of virus replication and the presence of inflammatory mediators in the plasma of patients and in specific tissues of animal models [36]. Interestingly, some cytokines secreted during alphavirus infection are the same of those associated with the progression of rheumatoid arthritis (RA), although inflammation in RA is clearly associated to an autoimmune process, which has not been consistently demonstrated for alphavirus-induced arthritis [48, 61]. Despite particular differences, expression analysis of inflammatory genes in a mouse model of CHIKV infection demonstrated similarities between the induced genes in this model and those induced in RA and collagen-induced arthritis models [61]. Furthermore, specific polymorphisms in human leukocyte antigen (HLA) as well as autoimmunity development, both conditions previously associated to patients' predisposition to rheumatic diseases and RA, were also observed in alphavirus-induced arthritis. The RA-associated alelles HLA-DRB1*01 and HLA-DRB1*04 were identified in CHIKV chronic patients [62]. These patients were later diagnosed for RA, and some of them were positive for autoantibodies, such as the rheumatoid factor (RF), anti-CCP (cyclic citrullinated peptide), and anti-nuclear antibodies, suggesting a role of CHIKV infection in RA initiation [62]. SINV infection also seems to be associated to HLA alleles involved in rheumatic diseases, in particular HLA-DRB1*01 [32, 63]. In addition, SINV-infected patients showed elevated titers of autoantibodies, including anti-nuclear and mitochondrial antibodies, with significant increase in RF three years postinfection [63]. Moreover, HLA-DR7 has been shown to be increased in patients with polyarthritis following RRV infection [64]. Taken together, these observations suggest that RA and alphavirus-induced arthritis share a set of common characteristics that could be useful in the development of therapeutic approaches against viral arthritis.

### 3.1. Role of the Target Cells for Alphavirus Replication in the Pathogenesis of Arthritis

Articular and nonarticular cells are involved in alphavirus replication and dissemination. Experimental models of alphavirus-induced arthritis suggest that pathogenesis results from a combination of a direct cellular and tissue damage caused by virus replication and an indirect immune response activation in target tissues [34, 37, 65]. Several cell types have been described as targets for arthritogenic alphavirus replication, including cells from joints, bones, and muscles as well as immune cells infiltrated in the synovium and in the infected tissues (Figure 1), highlighting the association between the tissues affected by virus replication and the local inflammatory process in the pathogenesis of alphavirus-induced arthritis.

SINV causes a persistent infection with periodic appearance of cytopathic effects in mouse fibroblasts cultures [66, 67]. In adult mice, SINV replicates in the periosteum, tendons, and endosteum of long bones [35]. Additionally, SINV has been isolated from a muscle biopsy of a patient with chronic myalgia and arthralgia 6 months after onset of the symptoms, indicating virus persistence in muscle cells [32]. This isolated virus was able to replicate in human myoblasts and myotubes cells *in vitro*, confirming virus tropism to muscle cells. Muscle necrosis accompanied by a massive infiltration of inflammatory cells has been observed in mouse models for RRV and CHIKV infection [34, 36, 68, 69]. Furthermore, CHIKV antigens were detected in skeletal muscle progenitor cells in patient biopsies during both the acute phase of CHIKV infection and the late recurrent symptomatic phase of the disease, with muscle necrosis and an inflammatory infiltrate observed in late phase [70]. The long lasting replication of RRV and CHIKV in muscle cells has been also supported by studies *in vitro* using primary mouse and human skeletal muscle cells, respectively [70, 71], reinforcing that viral replication in muscle cells is closely associated with acute and chronic myalgia observed in patients.

Macrophage has been described as the main component of cellular infiltrate observed in the injured tissues after alphavirus infection *in vivo *[34, 51]. The first evidence and the characterization of the central role of macrophage in arthritis pathogenesis have been demonstrated in studies with RRV. RRV antigens were detected in synovial monocytes/macrophages of patients after the beginning of the symptoms onset [47]. Furthermore, lineages of mouse monocytes/macrophages infected with RRV *in vitro* supported a continuous production of viruses for over 50 days after infection with restricted cytopathic effects [33, 72]. Additionally, pharmacological depletion of macrophages in mouse models of RRV and CHIKV infection resulted in lesser extent of muscular/articular damage, demonstrating the importance of macrophages for disease progression [33, 37, 73]. The ability of other alphaviruses besides RRV to replicate and persist in macrophages has also been demonstrated [74–76]. Primary human monocytes and macrophages infected with SINV and CHIKV showed a highly productive viral replication [75, 76]. In an immunocompetent nonhuman primate animal model of CHIKV infection, viral RNA was found 90 days postinfection mainly in spleen and lymph nodes, and macrophages appear to be the primary cells responsible for viral persistence in late stages of infection in this model [51]. Contribution of macrophages to the disease establishment may be due to an association between the maintenance of viral replication and the synthesis of inflammatory mediators in damaged tissue (Figure 1). Additionally, soluble factors secreted from macrophage can amplify the inflammatory process recruiting and activating lymphocytes and NK cells to target tissues [42, 49]. Thus, macrophages seem to be the most suitable candidate for viral reservoirs in affected tissues, playing a central role in alphavirus-induced arthritis. 

### 3.2. Immune Response and Inflammatory Mediators in Alphavirus-Induced Pathology

Several clinical, *in vivo,* and *in vitro* studies have been carried out to further elucidate the inflammatory process triggered by alphavirus infection and its participation in arthritis pathogenesis. 

To investigate the role of cellular immune response during alphavirus infection, several animal models of arthritis induced by RRV, CHIKV, or ONNV were developed. Severe inflammation was observed in bone, joint, and muscle tissues in a mouse model of RRV infection [34], and this inflammatory process was not altered in infected mice deficient in the recombinase activating gene (RAG^−/−^), which lack the functional T and B lymphocytes [34]. Furthermore, a recent study with adult RAG2^−/−^, CD4^−/−^, and CD8^−/−^ CHIKV-infected mice demonstrated that CHIKV-specific CD4^+^ but not CD8^+^ T cells are involved in joint swelling [77]. Together, these observations suggest that adaptive immune response has a restricted role in RRV and CHIKV disease pathology. In contrast, pharmacologic depletion of macrophages in mice infected with RRV resulted in the abrogation of disease symptoms and in a lower expression levels of IFN-*γ*, TNF-*α*, IL-*β*, MCP-1 and MIP-1*α* in muscle and joint tissues when compared to RRV-infected undepleted mice [38, 73]. Moreover, neutralization of IFN-*γ*, TNF-*α*, and MCP-1 reduced the clinical score of RRV-infected mice [73]. Similar effects of macrophages depletion was also evident in CHIKV infection, demonstrating a critical role of innate immunity in disease progression [37]. This was reinforced by the observation that CHIKV-infected patients who developed chronic symptoms showed an intense activation of several immune cells in the acute phase of the disease, including the DC, NK, CD4^+^, and CD8^+^ cells [31].

Infection by arthritogenic alphaviruses results in the production of a broad range of cytokines and chemokines, which were systematically detected through distinct experimental approaches (Table 2). The profile of these inflammatory mediators has been associated with the severity and persistence of infection. Proinflammatory mediators, such as IL-6, TNF-*α*, IFN-*α*/*β*, and IFN-*γ* were detected in the sera from RRV-infected and CHIKV-infected mice as well as CHIKV-infected nonhuman primates [37, 51, 73, 79]. The viremia phase was correlated to increased serum levels of several chemokines, such as MCP-1, RANTES, and IP-10, as well as an increase in their mRNA expression in the affected tissues [37, 51, 73, 79]. A strong local activation of the IFN-*γ* program was also demonstrated in the symptomatic phase of the disease [79]. In agreement with these observations, *in vitro *studies showed an increased expression of IL-8, MCP-1, and GM-CSF in synovial fibroblasts infected with RRV [78]. Consistently, CHIKV infection of a mouse macrophage lineage was associated with an enhanced production of TNF-*α*, IL-6, and GM-CSF [74]. In addition, primary human osteoblasts were shown to be susceptible to CHIKV infection *in vitro* and infection induced IL-6 and RANKL secretion by these cells with similar kinetics, while osteoprotegerin secretion was gradually inhibited [52]. Thus, infection of osteoblasts by CHIKV and the consequent IL-6 production may contribute to bone loss and to the occurrence of arthralgia and arthritis [52]. Interestingly, a comparison between CHIKV-induced and RA-induced gene expression in mouse models showed a remarkable similarity regarding the immune mediators, including IFNs, IL-4, IL-10, TNF-*α*, IL-15, GM-CSF, IL-8, and lymphotoxin B [61]. Furthermore, the overlap of gene expression profile between these two diseases increases with severity.

In a clinical study, CHIKV-infected patients in Singapore, the plasma levels of several cytokines and chemokines, including IFN-*α*, IL-6, IL-12, GM-CSF, IP-10 and MCP-1, correlate with the viral load, and plasma levels of IL-6 and GM-CSF were significantly increased in patients with persistent arthralgia [50]. In similar clinical studies, higher levels of IL-1*β*, IL-10, and IL-6 were also detected in patient sera, being IL-1*β* and IL-6 identified as biomarkers of disease severity and persistence [80]. In addition, IL-6 has been associated with the generation of joint pain [83], which reinforces the importance of this cytokine in the progression of disease. Besides cytokines, chemokines such as MCP-1, MIP-1*α*, and MIP-1*β* were increased during the chronic phase of CHIKV infection [81]. Elevated levels of MCP-1 were also found in RRV-infected patients [73]. On the other hand, low levels of RANTES were observed in severe and chronic patients [80, 81]. Another clinical study performed during a CHIKV outbreak in Italy showed that IL-6 and the chemokines CXCL9/MIG, MCP-1, and IP-10 were significantly increased in acute phase of disease [82]. In the same work, CXCL9/MIG, IP-10, and high titers of IgG were found in patients with mild and severe symptoms six months after initial infection when compared to recovered patients, suggesting that these factors may be used as disease severity markers [82]. These findings show again a remarkable similarity between alphavirus-induced arthritis and RA, in which CXCL9/MIG and IP-10 are also used as disease markers [84–88]. Also, IgG antibodies seem to be implicated in alphavirus infection as well as in RA, in which these antibodies act through the activation of the mast cells leading to synovial destruction and immune complex formation within the joint [89, 90].

MCP-1 levels are increased in patients in the majority of the clinical studies of alphavirus-induced arthritis [50, 73, 81, 82], suggesting an important role of this chemokine in recruitment of inflammatory cells to injured tissues. In CHIKV-infected patients MCP-1, IL-6, and IL-8 levels were higher in synovial fluids than in the sera, suggesting an active monocyte/macrophage trafficking into the synovial tissue. High levels of matrix metalloproteinase-2 (MMP2) were also found in the synovial tissue of one chronic patient, which would be one of the factors involved in tissue lesion [31]. In agreement, inhibition of MCP-1 action in animal models of RRV and CHIKV infection reduces inflammatory infiltrated, also supporting this hypothesis [91, 92]. MIF, a key cytokine in RA, has also been implicated in the exacerbation of the inflammatory process in RRV and SINV infection [65, 76]. In RA, MIF stimulates synovial macrophages to release several cytokines and the matrix metalloproteinases MPP1 and 3, contributing to tissue destruction in the joints [93, 94]. Likewise, we have demonstrated that SINV replication in human macrophages induced MIF, TNF-*α*, IL-1*β*, and IL-6 secretion, followed by an enhancing in the expression of MMP1 and 3, and that cytokine secretion and MMP expression were primarily regulated by MIF [76]. Additionally, RRV infection of MIF-deficient mice caused a mild disease when compared to that developed in wild-type animals, with inflammatory infiltrate reduction accompanied by a lower expression of MCP-1 and IFN-*γ* in muscle and joints, leading to a decrease in muscle tissue destruction, although the viral titers were similar [65]. As expected, RRV-infected wild-type mice treated with recombinant MIF developed more pronounced disease signs.

### 3.3. Involvement of the Complement Cascade in the Arthritis Caused by Alphaviruses

Complement activation was detected in the synovial fluids of RRV-infected patients. Levels of C3a, a marker of the central complement system C3 processing, were higher in RRV-infected patients than in patients with noninflammatory osteoarthritis [95]. In agreement with these observations, recent findings obtained using a mouse model of RRV-induced arthritis showed that complement is important to promote inflammatory tissue destruction [95]. Besides the detection of the complement activation products in the serum and in the inflamed joints and muscles of RRV-infected wild-type mice, RRV-infected C3-deficient mice (C3^−/−^) developed a less severe disease and also presented much lower levels of skeletal muscle destruction, despite having similar inflammatory infiltrates than RRV-infected wild-type mice [95].

 C3 receptor (CR3 or CD11b/CD18, Mac-1, *α*_*m*_ *β*_2_) binds several different ligands, including iC3b, a C3 cleavage fragment. As observed for C3^−/−^ mice, RRV-infected CR3-deficient mice (CD11b^−/−^) develop a less severe disease and lower tissue destruction when compared to RRV-infected wild-type mice [96]. CR3 deficiency had no effect on viral replication and inflammatory infiltration, but the expression of the proinflammatory proteins S100A9, S100A8, and IL-6 were significantly reduced in RRV-infected C3^−/−^ and CD11b^−/−^ mice when compared to RRV-infected wild-type mice [96]. In agreement, the levels of heterodimeric complex formed by S100A9 and S100A8 were elevated in the sera of patients with RA or inflammatory muscle diseases, in which the expression of these proteins by macrophages had been associated with muscle fibers degeneration [97–99].

The complement activation pathways that are determinant for the pathogenesis of RRV infection in mice were identified using deficient mice for the key components of the classical (Clq^−/−^), alternative (factor B, Fb^−/−^), or mannose binding lectin (MBL^−/−^) pathways [100]. RRV-infected MBL^−/−^ mice developed less pronounced disease signs, with reduced tissue damage and C3 deposition in muscle tissues. On the other hand, infected Clq^−/−^ and fB^−/−^ mice presented normal disease progression and severity [100]. These observations suggest that RRV infection leads to complement activation through MBL pathway, which contributes to RRV disease severity. In RRV-infected patients, higher MBL levels in both serum and synovial fluid correlated with polyarthritis severity [100], reinforcing the importance of MBL pathway.

### 3.4. Role of Alphavirus Evasion from Host Antiviral Defense in Pathogenesis

Type I IFN immune response signaling is essential for the control of viral replication and could be the key process in preventing virus dissemination toward the target tissues and the development of alphavirus-induced arthritis. Indeed, IFN-stimulated genes (ISGs) are critical in controlling CHIKV, RRV, SINV, and ONNV replication [17, 101–103]. In a mouse model of ONNV infection, deficiency in STAT, which couples IFN signaling, increases disease lethality [17]. Mice deficient in type I IFN were more susceptible to CHIKV infection, with a broader dissemination of the virus, which reaches the central nervous system besides replicating in liver, muscles, and joints [54]. Viperin, product of an ISG, has been also shown to be critical for host antiviral response to CHIKV infection. Viperin expression, together with type I IFNs and some related ISGs expression, was highly induced in PBMCs of CHIKV-infected patients with a viral load-dependent profile, and CHIKV-infected mice deficient in viperin showed an enhanced viral load and a more severe joint inflammation when compared to infected wild-type mice [104]. Studies using samples from a cohort CHIKV-infected patients showed a tight association between high viral load and an enhanced expression of IFN-*α*/*β* and several genes of the type I IFN signaling pathway, such as IRF3, IRF7, and RSAD2 (viperin encoding gene), in patients PBMCs [104]. Furthermore, CHIKV infection activates directly IRF3, inducing the transcription of IFN-*β* itself and several ISGs through the activation of IPS-1 [105]. In SINV infection, the induction of type I IFN expression was also dependent on the activation of IRF3, which occurs through the host intracellular pattern recognition receptor (PRR) MDA5 [106]. RRV has been also shown to be recognized by PRR: mice deficient in Myd88 or TLR7 genes infected with RRV develop more extensive tissue damage and higher viral titers than infected wild-type mice [107]. TLR7-deficient mice also produce elevated levels of RRV specific antibodies but with little neutralizing activity and lower epitope affinity when compared to RRV specific antibodies produced by wild-type mice [107]. CHIKV clearance seems to be dependent on both RIG-like receptors and TLRs, which trigger a type I IFN response that acts directly in nonhematopoietic cells, controlling CHIKV replication in the local of infection and preventing virus dissemination [108]. 

Despite inducing IFN production, arthritogenic alphaviruses are able to antagonize type I IFN response (Figure 1). SINV replication bypasses the need of a functional IFN-induced phosphorylated eiF2*α* for translation, using an alternative pathway to locate the ribosomes on the initiation codon of the viral RNA [109]. Although CHIKV induces ISGs expression, it promotes a widespread translation shutoff of cellular genes through eiF2*α* phosphorylation by PKR, while the translation of viral proteins is maintained. [105]. In late infection, CHIKV also induces transcription shutoff of IFN-*β* and ISGs. In addition, the nonstructural protein nsP1 antagonizes the action of the ISG BST-2 (bone marrow stromal antigen 2, a protein impairs CHIKV particles budding from the infected cells) [110]. 

Several alphaviruses' virulence factors are involved in viral persistence and evasion from the immune system. Mice deficient in STAT1-dependent IFN response infected with CHIKV developed a much more severe muscoloskeletal pathology with an increased viral replication in joint-associated tissues when compared to infected wild-type mice [111], supporting the hypothesis that alphaviruses' ability to inhibit the IFN-induced JAK/STAT signaling pathway is related to their virulence *in vivo*. Also, infection of adult mice deficient in IRF3 and IRF7 with CHIKV is lethal, and mortality has been associated with an increased virus replication and pathogenesis [112]. 

Genetic determinants in viral nonstructural proteins nsP1 and nsP2 were also associated to the modulation of STAT activation and to the virulence in SINV and RRV [113, 114]. Additionally, SINV nsP2 has been implicated in the development of the cytopathic effect induced by infection [115]. Furthermore, small-plaque mutant RRV (with mutations in E2 and nsP regions) showed increased resistance to IFN*α*/*β* antiviral response compared to the parental strain, which allows high virus titers in mice, leading to an increase in the severity of hind limb disease, myositis, and mortality [116]. 

The induction of type I IFN response by RRV is also dependent on whether the virus is produced by mammalian or mosquitos cells. The mosquito cell-derived virus fails to induce IFN*α*/*β* due to the lack of complex carbohydrates on virus particle, and it seems that N-linked glycans in E2 glycoprotein from the mammalian-cell-derived virus are needed for a strong IFN response [117, 118]. 

Altogether, these findings suggest that viremia control in alphavirus infection depends on different factors such as the presence of strain virulence determinants in nsP1 and nsP2, the extent of the induction of type I IFN response during infection as well as the virus ability to evade from this response. Since IFN response is activated early in the disease, viral persistence in affected tissues during chronic phase of arthritis might be seen as a failure in this early response.

## 4. Concluding Remarks

Even with the recent advances in the understanding of the pathogenesis of joint damage associated with alphavirus infection, many gaps remain and need to be explored. Most of the studies are currently focused on CHIKV infection and therefore the differences and similarities among the mechanisms involved in arthropathy induction by the distinct alphaviruses still cannot be pointed out. Improvements in the diagnostic of new cases as well as in the generation of animal models for the study of the arthritis induced by SINV and MAYV consist in a key challenge for the progress in a broader understanding of the mechanisms involved in alphavirus-induced arthritis. 

The data accumulated so far indicate that the pathogenesis involved in alphavirus-induced joint damage is determined by host inflammatory response as well as by virus persistence and virulence. Inflammatory response includes the production of cytokines, chemokines, and other inflammatory mediators that are involved in macrophage, NK, and T cells recruitment to the sites of viral replication (Figure 1). Viral persistence could occur in target tissue, as muscles and joint connective tissues, but macrophages seem to be the main viral reservoirs and may play an important role in virus dissemination to the target tissues. Chronic infection of host cells is also closely related to the chronic disease establishment and the long lasting of the symptoms. Furthermore, differences in alphavirus genetic determinants promote virulence and evasion from the cellular antiviral response, which may contribute to disease development.

Some efforts have been made toward the development of therapeutic approaches against alphavirus-induced arthritis. Drugs used to control inflammation in patients with RA have been used as supportive therapy to joint symptoms in patients infected with RRV and CHIKV, but the results were limited and variable [28, 69, 119]. Mouse models for RRV and CHIKV infections have been useful to test drugs that control host inflammatory response, such as bindarit, an inhibitor of MCP-1 receptor [91, 92]. Nonetheless, the understanding of the mechanisms involved in the pathogenesis of alphavirus-induced arthritis as well as the establishment of novel animal models are essential steps to the development and characterization of new molecular targets and more efficient and selective drugs to treat these diseases.

## Figures and Tables

**Table 1:** Occurrence and geographic distribution of arthritogenic alphaviruses.

Virus	First description	Geographic distribution	Occurrence	References
RRV	1928, in New South Wales, Australia	Australia, Papua New Guinea, Solomon Islands, and the South Pacific Islands	Endemic in Australia and Papua New Guinea, annual epidemics in Australia (~4,000 cases per year). Major epidemics: ~60,000 cases in 1979 in Pacific Islands ~8,000 cases in 1996 in Australia	[2, 4, 23]

SINV	1952, in Sindbis village, near Cairo, Egypt	Europe, Asia, Africa, and Oceania.	Endemic in North Europe; Outbreaks in Finland, Norway, Sweden and Russia (late summer or early autumn)	[2, 4, 21]

CHIKV	1952, in Newala, Tanzania	Africa and Asia (documented cases in Europe, USA, and Oceania)	Sporadic epidemics in Africa and Asia, imported cases reported in Europe and USA. Major epidemics: ~300,000 cases in 2006-2006 in La Réunion (French Indian Ocean territory) ~1.4–6.5 million cases in 2006-2007 in India	[4, 59, 60]

MAYV	1954, in Trinidad and Tobago	Northern South America	Endemic in tropical regions of South America Sporadic outbreaks Pan-Amazonia forest regions	[4, 8, 19]

ONNV	1959, in northern Uganda	Africa	Rare epidemics in Africa (disappeared for 35 years from 1961 to 1996) ~2 million cases in 1959–1961 in East Africa	[2, 4]

BFV	1974, in the Barmah Forest, Australia	Australia	Annual epidemics in Australia (~1,000 cases per year)	[4, 25]

**Table 2:** Inflammatory mediators in arthritogenic alphaviruses infection.

Virus	Cell cultures infected *in vitro *	Animal models	Patients		References
			Acute phase	Chronic phase	
RRV	IL-8, GM-CSF, MCP-1	MIF, MCP-1, MIP-1*α*, TNF-*α*, IL-1*β*, IFN-*γ*	TNF-*α*, IFN-*γ*, MCP-1		[65, 73, 78]
CHIKV	IL-6, TNF-*α*, GM-CSF, MCP-1	IFN-*α*/*β* IFN-*γ*, KC, MCP-1, IP-10, IL-6, IL-10, IL-1*β*, TNF-*α*, IL-15, GM-CSF	IL-6, IFN-*α*, IP-10, IL-12, IL-1Ra, MCP-1, IL-10, IL-15, MIG	IL-6, GM-CSF, IL-1*β*, IL-8, IL-1Ra, MCP-1, MIP-1*α*, MIP-1*β*	[31, 37, 50–52, 61, 74, 79–82]
SINV	IL-6, TNF-*α*, IL-1*β*, MIF				[76]
